# Structure-Based Vaccines Provide Protection in a Mouse Model of Ehrlichiosis

**DOI:** 10.1371/journal.pone.0027981

**Published:** 2011-11-17

**Authors:** Sunil Thomas, Nagaraja R. Thirumalapura, Patricia A. Crocquet-Valdes, Bruce A. Luxon, David H. Walker

**Affiliations:** 1 Department of Pathology, University of Texas Medical Branch, Galveston, Texas, United States of America; 2 Center for Biodefense and Emerging Infectious Diseases, University of Texas Medical Branch, Galveston, Texas, United States of America; 3 Institute of Human Infections and Immunity, Institute for Translational Science, Department of Biochemistry and Molecular Biology, University of Texas Medical Branch, Galveston, Texas, United States of America; 4 Sealy Center for Vaccine Development, University of Texas Medical Branch, Galveston, Texas, United States of America; Federal University of São Paulo, Brazil

## Abstract

**Background:**

Recent advances in bioinformatics have made it possible to predict the B cell and T cell epitopes of antigenic proteins. This has led to design of peptide based vaccines that are more specific, safe, and easy to produce. The obligately intracellular gram negative bacteria *Ehrlichia* cause ehrlichioses in humans and animals. As yet there are no vaccines to protect against *Ehrlichia* infection.

**Methodology/Principal Findings:**

We applied the principle of structural vaccinology to design peptides to the epitopes of *Ehrlichia muris* outer membrane P28-19 (OMP-1/P28) and *Ehrlichia* Heat shock protein 60 (Hsp60/GroEL) antigenic proteins. Both P28-19 and *Ehrlichia* Hsp60 peptides reacted with polyclonal antibodies against *E. canis* and *E. chaffeensis* and could be used as a diagnostic tool for ehrlichiosis. In addition, we demonstrated that mice vaccinated with *Ehrlichia* P28-19 and Hsp60 peptides and later challenged with *E. muris* were protected against the pathogen.

**Conclusions/Significance:**

Our results demonstrate the power of structural vaccines and could be a new strategy in the development of vaccines to provide protection against pathogenic microorganisms.

## Introduction

Vaccines are considered as one of the most successful medical intervention against infectious diseases. Vaccines include killed or attenuated organisms or purified products derived from them. One of the drawbacks of killed or attenuated vaccines is the potential side effect of some of the antigenic proteins. This led to the design of recombinant vaccines based on whole antigens. As whole antigenic proteins are not essential in inducing immunity, it led to the emergence of a new branch of vaccine design termed structural vaccinology [1], [2]. Structure based vaccines have the rationale that protective epitopes are enough to induce immune responses and provide protection against pathogens [3]. Structure-based peptide antigens induce antibodies which recognize the denatured form of a protein from which their sequences are derived [4].

The obligate intracellular bacterium *Ehrlichia chaffeensis* that resides in mononuclear phagocytes is the etiologic agent of human monocytotropic ehrlichiosis (HME). HME is an emerging and often life-threatening zoonotic, tick-transmitted infectious disease in the United States [5]–[7]. Lack of early diagnosis and treatment of HME are the main factors that lead to severe and fatal disease. *Ehrlichia* also causes diseases in companion animals and domesticated ruminants. *E. chaffeensis* and *E. canis* cause canine ehrlichioses in dogs, whereas *E. ruminantium* causes heartwater in cattle, sheep and goats. Vaccines are needed for these tick transmitted pathogens, but are hindered by many obstacles that exist in their development. These include knowledge of genetic and antigenic variability, identification of the ehrlichial antigens that stimulate protective immunity or elicit immunopathology, development of animal models that reflect the immune responses of the hosts and understanding molecular host-pathogen interactions involved in immune evasion or that may be blocked by the host immune response. As yet there are no commercially available vaccines to protect against ehrlichiosis [8].

Development of a murine model of persistent ehrlichiosis has greatly facilitated our understanding of the pathogenesis and mechanisms of host defenses against ehrlichial infections. Mildly virulent *Ehrlichia muris* infection in immunocompetent C57BL/6 mice results in persistent infection and mimics *E. chaffeensis* infection in its natural host, white-tailed deer [9]. Murine models of systemic infection associated with the mildly virulent *E. muris* or the highly virulent IOE (*Ixodes ovatus Ehrlichia)* have provided knowledge of immunological mechanisms involved in host defenses against ehrlichial infection [9]–[15]. Protective immunity against *E. muris* in the mouse models of ehrlichiosis correlates with induction of strong cell-mediated CD4 and CD8 type 1 responses and humoral immunity [12]. T cell independent humoral immunity has also been reported to be sufficient for protection against fatal infection with intracellular ehrlichial pathogens [16].

We recently demonstrated that *Ehrlichia* Hsp60 (GroEL) and P28 (OMP-1) are the major antigenic proteins of *E. muris* and they are also post-translationally modified [17]. Based on these observations, we bioinformatically identified the predicted hydrophilic epitope sequences of *Ehrlichia* Hsp60 and P28-19 and synthesized peptides to use as diagnostic probes to detect antibodies against *Ehrlichia.* In this paper we demonstrate that both *Ehrlichia muris* Hsp60 and P28 peptides reacted with antibodies against *E. chaffeensis* and *E. canis* and could be used in diagnosis of infected animals. We also demonstrated that the structure-based peptides provided protection against *Ehrlichia* and hence could be used as a vaccine against ehrlichioses.

## Results

### Peptides based on the epitopes of *Ehrlichia* Hsp60 and outer membrane protein P28-19 detected pathogen-specific antibodies

The outer membrane proteins P28 and *Ehrlichia* Hsp60 (GroEL) are the major antigenic proteins of *Ehrlichia* [17]. Previous studies in our laboratory and elsewhere have demonstrated that P28-19 is the most efficient and protective P28 paralog which provides protection against *Ehrlichia* [18], [19].

**Figure 1:**
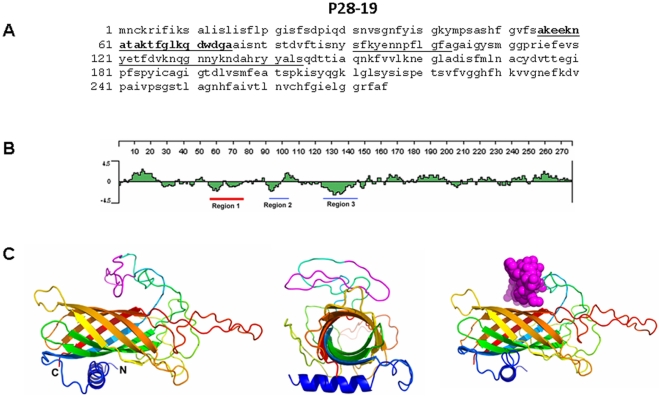
Amino acid sequence of P28-19. (A) P28-19 peptides corresponding to the underlined predicted hydrophilic sequence were synthesized. The peptide corresponding to the bold underlined (55–75) sequence was found to react with antibodies to *Ehrlichia* as well as to induce antibody production. (B) Hydrophobicity plot of P28-19. The sequences underlined (in red and blue) were used for synthesizing peptides; however the best peptide sequence selected is underlined in red. (C) (Left) Predicted 3D structure of P28-19 (side view), (Middle) predicted 3D structure of P28-19 (basal view), (Right) predicted 3D structure of P28-19 with the Van der Waals radii of the heavy atoms highlighting the region of interest (P28-19 _55−75_).

We identified the *Ehrlichia* Hsp60 and P28-19 epitopes based on the predicted hydrophilicity [20]. Three regions of the *E. muris* P28-19 protein sequence had substantial predicted hydrophilicity as determined by the Lasergene software. The peptides correspond to amino acids 55–75, 91–103, and 124–145 (Fig. 1). Initial studies demonstrated that the peptide corresponding to the amino acids 55–75 of the P28-19 protein is more sensitive in detection of *Ehrlichia* specific antibodies. Based on the information we injected P28-19 _55−75_ peptide to induce antibody generation or function as a vaccine candidate.

**Figure 2:**
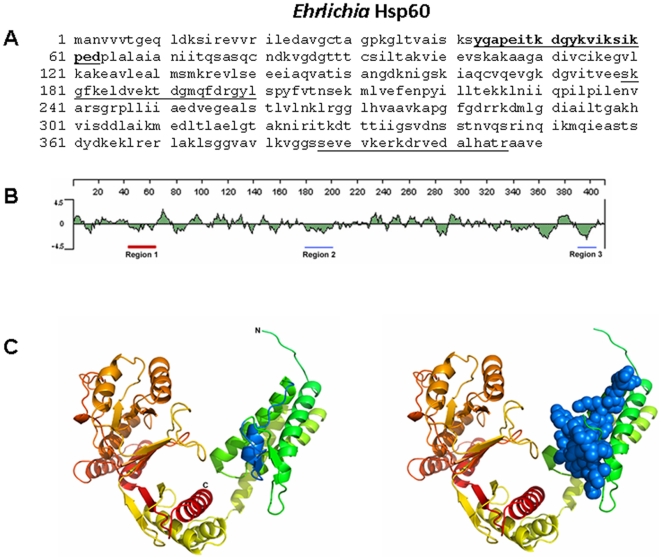
Amino acid sequence of *Ehrlichia* Hsp60. (A) Hsp60 peptides corresponding to the underlined predicted hydrophilic sequence were synthesized. The peptide corresponding to the bold underlined (43–63) sequence was found to react with antibodies to *Ehrlichia* as well as to induce antibody production. (B) Hydrophobicity plot of *Ehrlichia* Hsp60. The sequences underlined (in red and blue) were used for synthesizing peptides; however, the best peptide sequence selected is underlined in red. (C) (Left) Predicted 3D structure of *Ehrlichia* Hsp60, (Right) predicted 3D structure of *Ehrlichia* Hsp60 with the Van der Waals radii of the heavy atoms highlighting the region of interest (Hsp60 _43–63_).

Three regions of the *Ehrlichia* Hsp60 protein sequence had substantial predicted hydrophilicity as determined by the Lasergene software (DNAStar, WI, USA). The peptides correspond to amino acids 43–63, 179–200, and 386–406 (Fig. 2). The peptide corresponding to amino acid 43–63 of *Ehrlichia* Hsp60 was found to be more reactive with various *Ehrlichia*- specific antibodies and also induced production of antibodies (Fig. 2). Based on the information we injected *Ehrlichia* Hsp60 _43–63_ peptide to induce antibody generation or function as a vaccine candidate.

**Figure 3:**
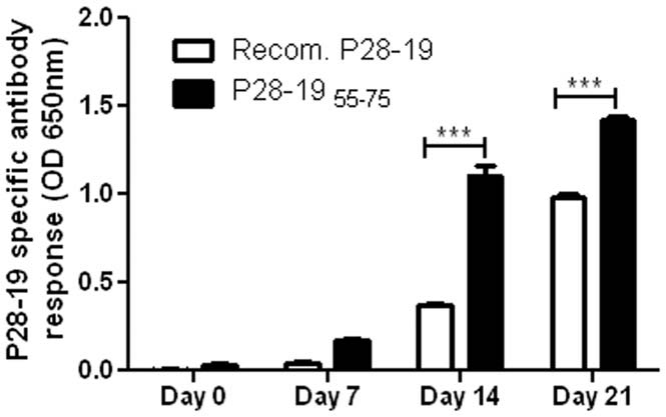
P28-19 _55-75_ peptide reacted with *E. muris* antibody. The peptide corresponding to the predicted hydrophilic sequence of amino acids 55–75 of P28-19 reacted with *Ehrlichia* antibody. The peptide was found to be more sensitive in reacting with the *Ehrlichia* antibody than the recombinant P28-19 protein (***p<0.001 as determined by Student *t* test).

We probed for *Ehrlichia*-specific antibodies in sera of mice infected with *E. muris*. As a control we used recombinant P28-19 protein. The peptide P28-19 _55–75_ was found to be more sensitive in detecting *Ehrlichia*-specific antibody than recombinant P28-19. The peptide detected the *Ehrlichia-*specific antibody even from sera of mice obtained as early as seven days after *E. muris* infection (Fig. 3). In subsequent studies we used P28-19 _55-75_ as a diagnostic probe or vaccine candidate.

**Figure 4:**
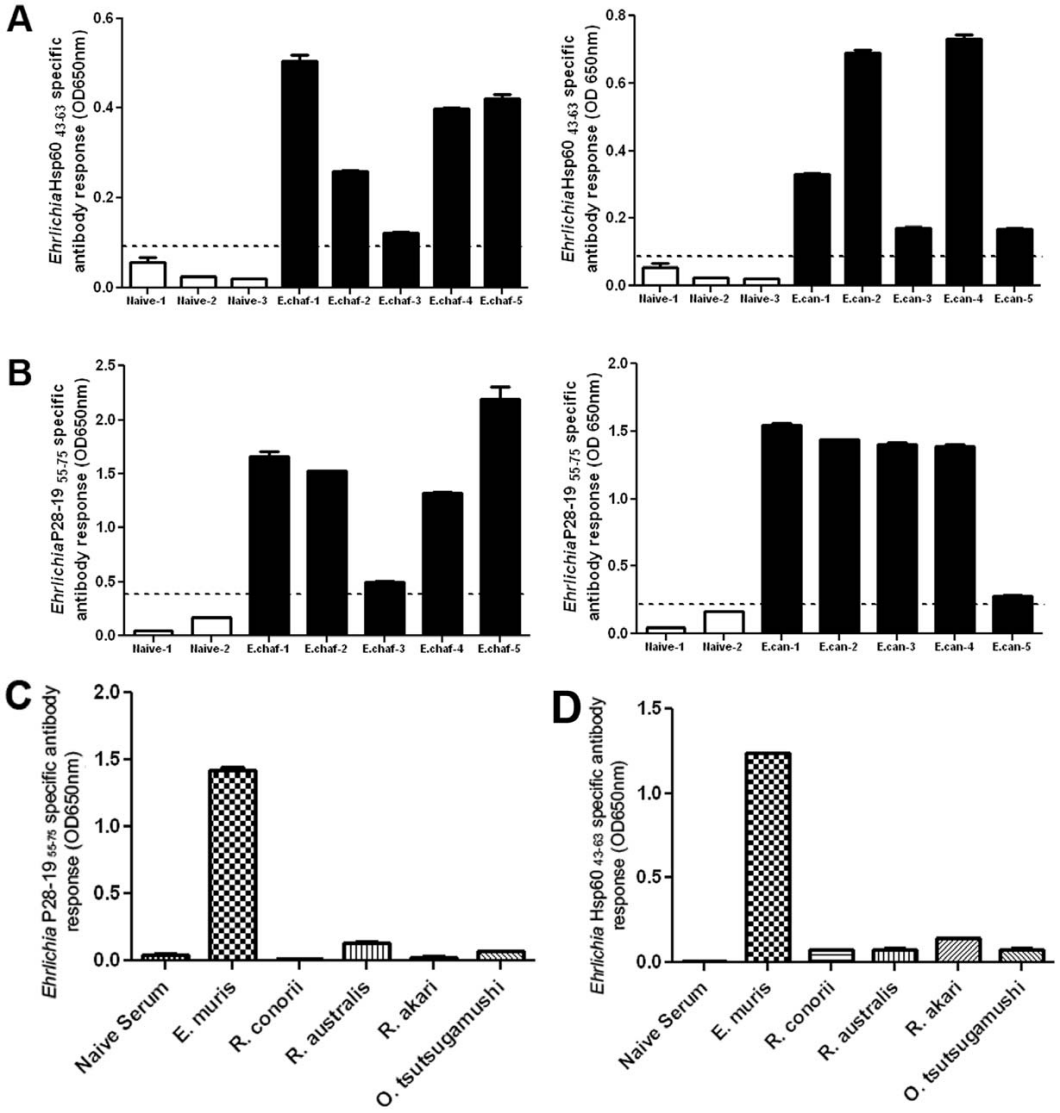
*Ehrlichia* Hsp60 _43–63_ and P28-19 _55–75_ peptides reacted with *Ehrlichia*-specific antibody from dogs infected with *E. canis* and *E. chaffeensis*. (A) *Ehrlichia* Hsp60 _43–63_ peptide reacted with antibodies from five dogs infected with *E. chaffeensis* and five dogs infected with *E. canis.* (B) P28-19 _55–75_ peptide reacted with antibodies from five dogs infected with *E. chaffeensis* and five dogs infected with *E. canis*. Each bar represents the mean of three replicates. The horizontal line in the graphs represents Mean + 3 SD of negative samples. The positive samples are significantly different from negative samples. (C) P28-19 _55–75_ peptide did not react with antibodies from mice infected with *Rickettsia* or *Orientia*. (D) *Ehrlichia* Hsp60 _43–63_ peptide did not react with antibodies from mice infected with *Rickettsia* or *Orientia*.

We probed sera of dogs with ehrlichioses to determine the efficacy of both P28-19 _55–75_ and Hsp60 _43–63_ peptides as diagnostic tools. We coated the peptides on an ELISA plate and assayed for *E. chaffeensis* and *E. canis* antibody from sera of infected dogs. The ELISA plate coated with the Hsp60 _43–63_ peptide detected *Ehrlichia-*specific antibody in the sera of dogs infected with *E. canis* or *E. chaffeensis* (Fig. 4A). The assay confirmed that the peptide corresponding to the *Ehrlichia* Hsp60 _43–63_ epitope could be used in diagnostics. Similarly, P28-19 _55–75_ peptide detected antibodies to both *E. chaffeensis* and *E. canis* from infected dog serum samples, whereas, there was no antibody binding from sera of uninfected dogs (Fig. 4B). To confirm that P28 and *Ehrlichia* Hsp60 peptides are specific to detect *Ehrlichia* but not other pathogens we probed sera of mice infected with *Rickettsia australis*, *R. conorii, R. akari* and *Orientia tsusugamushi.* The P28-19 _55-75_ and *Ehrlichia* Hsp60 _43–63_ peptides did not react with sera of mice infected with *Rickettsia australis*, *R. conorii, R. akari* and *Orientia tsusugamushi* (Fig. 4C, 4D). The experiments demonstrated that both *Ehrlichia* Hsp60 _43–63_ and P28-19 _55–75_ epitope peptides could be used as antigens in diagnostic applications.

### Antibodies against the epitopes of *Ehrlichia* Hsp60 and the outer membrane protein P28-19 detected pathogen-specific antigen

**Figure 5:**
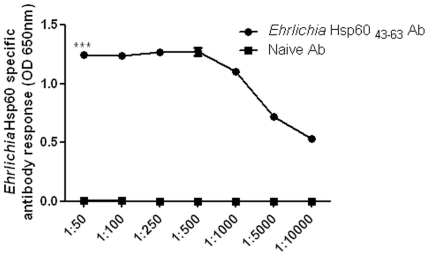
Reactivity of *Ehrlichia* Hsp60 antibody. The *Ehrlichia* Hsp60 _43–63_ peptide was sensitive in detecting different dilutions of the *Ehrlichia* Hsp60 antibody. The *Ehrlichia* Hsp60 (250ng) was probed with different dilutions of *Ehrlichia* Hsp60 specific sera or sera from naïve mice. Finally they were probed with goat anti-mouse-AP (1∶2000) and the OD measured at 650 nm after the addition of substrate. Each value is the mean of 3 replicates (***p<0.001 as determined by two way ANOVA).

**Figure 6:**
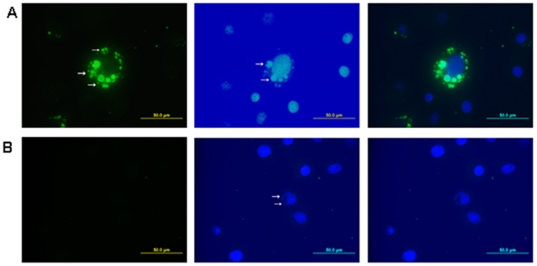
Detection of *E. muris* by fluorescence microscopy. (A) Antibodies produced against peptide P28-19 _55–75_ detected *E. muris* in infected DH82 cells. Left: Infected DH82 cell probed with P28-19 antibody, followed by FITC conjugated goat anti-mouse antibody. Middle: DAPI staining. Right: Merge. Arrows indicate *E. muris.* (B) Naïve serum did not detect *E. muris* in DH82 cells. Left: Infected DH82 cell probed with naive serum, followed by FITC-conjugated goat anti-mouse antibody. Middle: DAPI staining. Right: Merge. Arrows indicate *E. muris.*

Antibodies are used extensively as diagnostic tools in a wide array of different analyses. We injected different amounts of *Ehrlichia* Hsp60 _43–63_ to induce polyclonal antibody. Fifty micrograms (0.02 µM) of *Ehrlichia* Hsp60 _43–63_ peptide induced the optimum amount of antibody for our studies (data not shown). Using ELISA we determined the detectable level of antibody which could bind to *Ehrlichia* Hsp60 _43–63_ peptide. The antibody to *Ehrlichia* Hsp60 detected *Ehrlichia* Hsp60 _43–63_ peptide even at a high dilution (Fig. 5) (*p*<0.001 as determined by two way ANOVA). We also used the antibody to probe for *E. muris* and *E. chaffeensis* in DH82 monocytic cells [21]. To determine whether the antibodies against the epitope of P28-19 _55–75_ could be used as a probe we immunized mice with P28-19 _55–75_ peptide and used the sera as a probe to detect *E. muris* in DH82 cells. The P28-19 _55–75_ peptide induced antibody production that detected *E. muris* in DH82 cells as determined by fluorescence microscopy (Fig. 6A).

### The peptides of P28-19 _55–75_ and *Ehrlichia* Hsp60 _43–63_ functioned as vaccines to protect against the pathogen

**Figure 7:**
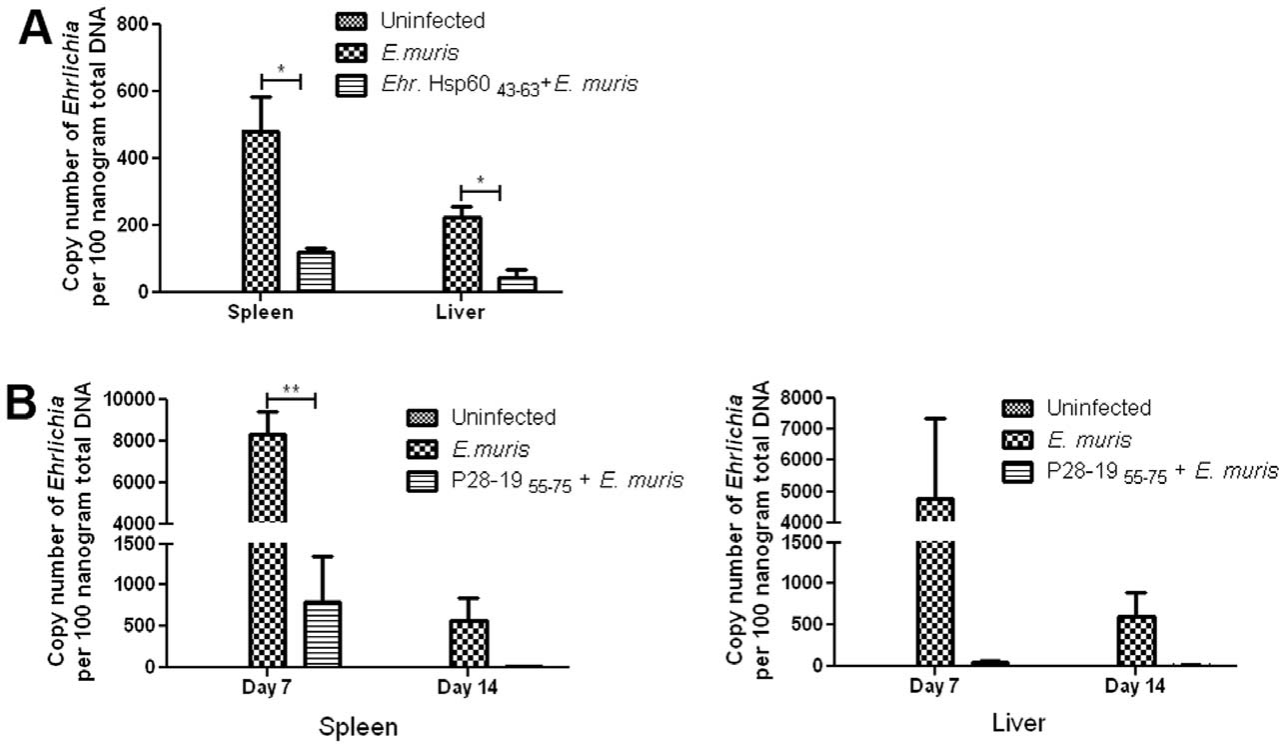
Immunization with *Ehrlichia* Hsp60 _43–63_ and P28-19 _55–75_ peptides protected mice from *Ehrlichia* infection. (A) Mice immunized with *Ehrlichia* Hsp60 _43–63_ were protected against *E. muris* challenge as determined by the bacterial load measured by quantitative real time-PCR on day 14 after *E. muris* challenge (*p<0.05 as determined by t test). (B) Mice immunized with P28-19 _55–75_ peptide was protected against *E. muris* challenge as determined by the bacterial load measured by quantitative real time-PCR on days 7 and 14 after *E. muris* challenge (**p<0.01 as determined by *t* test).

Since the P28-19 _55–75_ and *Ehrlichia* Hsp60 _43–63_ epitope peptides induced antibodies, we reasoned that they also could provide protection against *Ehrlichia* thereby functioning as potential vaccine candidates. To prove our hypothesis mice were injected with P28-19 _55–75_ or *Ehrlichia* Hsp60 _43–63_ epitope peptides and challenged 30 days later with *E. muris*. The spleen and liver were collected at different days after bacterial challenge and the bacterial copy number determined by quantitative real time PCR. We observed lower bacterial load in both spleen and liver on days 7 and 14 after bacterial infection in the vaccinated mice compared to unvaccinated controls (Fig. 7). The results demonstrated that P28-19 _55–75_ and *Ehrlichia* Hsp60 _43–63_ peptides functioned as vaccine candidates and provided protection against *Ehrlichia* infection.

**Figure 8:**
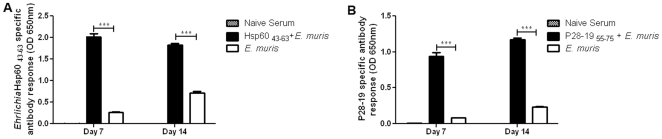
Protection induced by *Ehrlichia* Hsp60 _43–63_ and P28-19 _55–75_ peptides was associated with induction of *Ehrlichia*- specific IgG antibody. (A) *Ehrlichia* Hsp60 _43–63_ vaccinated mice induced higher IgG antibody levels after challenge with *E.* muris compared to unvaccinated *E. muris*-infected mice (***p<0.001 as determined by t test). (B) P28-19 _55–75_ peptide vaccinated mice induced higher IgG antibody levels after *E. muris* challenge compared to unvaccinated *E. muris-*infected mice (***p<0.001 as determined by Student *t* test).

Immunization with vaccines stimulates the immune system to produce a robust antibody response that can provide protection against pathogens. To determine the antibody responses against the *Ehrlichia* Hsp60 _43–63_ peptide vaccine, we collected blood from vaccinated mice on days 7 and 14 and performed ELISA. There was a significant difference in the antibody response between unvaccinated and *Ehrlichia* Hsp60 _43–63_ vaccinated mice after challenge with *E. muris*. However, there was no difference between the antibody levels in vaccinated mice between days 7 and 14. The *Ehrlichia* Hsp60 _43–63_-specific antibody levels in infected unvaccinated mice were highest on day 14 compared to day 7 (Fig. 8A). To determine the antibody responses against the P28-19 _55–75_ peptide vaccine, we collected blood from immunized mice on days 7 and 14 and subjected the samples to ELISA. There was a significant difference in the antibody response between unvaccinated and P28-19 _55–75_ vaccinated mice after challenge with *E. muris*. Antibody levels were higher on day 14 compared to day 7 (Fig. 8B).

**Figure 9:**
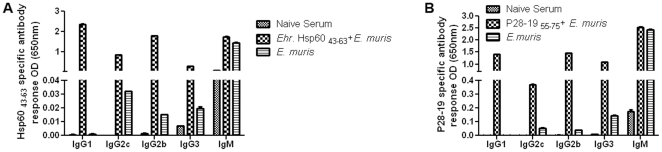
Antibody isotypes in mice immunized with *Ehrlichia* Hsp60 _43–63_ and P28-19 _55–75_ peptides. (A) Mice vaccinated with *Ehrlichia* Hsp60 _43–63_ peptide had higher levels of IgG1, IgG2c, IgG2b, and IgG3 compared to unvaccinated mice after bacterial challenge. (B) Mice vaccinated with P28-19 _55–75_ peptide had higher levels of IgG1, IgG2b, IgG2c, and IgG3 compared to unvaccinated mice after bacterial challenge. The data were expressed as mean plus standard deviation and three mice per group were included for analysis.

As antibody isotype responses can be useful indicators of immune bias during infection [22], we determined the antibody isotypes after vaccination with the peptide epitopes. The level of antibody isotypes increased by day 14 compared to day 7 after bacterial challenge (data not shown). The *Ehrlichia* Hsp60 _43–63_-vaccinated mice had higher levels of IgG1, IgG2c, IgG2b, IgG3 and IgM after bacterial challenge compared to unvaccinated mice on day 14 (Fig. 9A). By ELISA we analyzed the isotypes of the antibodies of P28-19 peptide in vaccinated and unvaccinated mice after challenge with *E. muris* (day 14 post challenge). The P28-19 _55–75_ vaccinated mice challenged with *E. muris* had higher levels of IgG1, IgG2b, IgG3 and IgM compared to unvaccinated mice infected with the pathogen (Fig. 9B).

### *Ehrlichia* Hsp60 and P28-19 specific memory CD4+ Th1 responses are induced during *E. muris* infection

**Figure 10:**
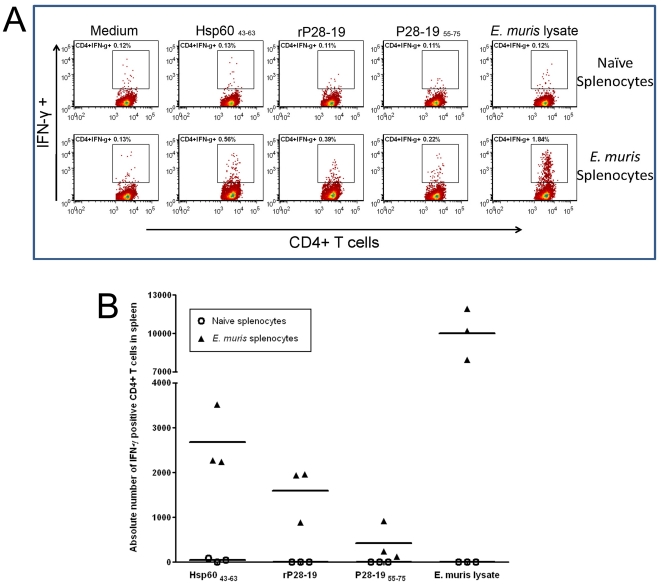
*Ehrlichia* Hsp60 _43–63_ and P28-19 _55–75_ -specific memory CD4+ T cells develop during *E. muris* infection. We determined by flow cytometry the frequencies and absolute numbers of *Ehrlichia* Hsp60 _43-63_- and P28-19–specific IFN-γ-producing CD4+ T cells in the spleen of mice infected with *E. muris*. (A) Mice infected with *E. muris* had higher frequency of *Ehrlichia* Hsp60 _43–63_- and P28-19 _55–75_ -specific IFN-γ-producing CD4+ T cells in the spleen on day 45 after infection compared to naïve uninfected mice. Representative dot plots were gated on live cells followed by CD3+ T cells (B) Absolute numbers of *E. muris*-specific IFN-γ-producing CD4+ T cells in the spleen of the same mice detected following *in vitro* stimulation with the *Ehrlichia* Hsp60 _43–63,_ P28-19 _55–75_ peptides; rP28-19 and *E. muris* whole cell lysate are shown for comparison. Horizontal bars represent the mean; data are representative of two independent experiments (n  = 3 animals per group).

We determined by flow cytometry if *Ehrlichia* Hsp60 _43–63_ and P28-19 specific memory T cells are induced during *E. muris* infection. Splenocytes from *E. muris*-infected mice were harvested on day 45 post-infection and stimulated *in vitro* with the *Ehrlichia* Hsp60 _43–63_ and P28-19 _55–75_ for 18h. Compared to uninfected naïve mice, *E. muris*-infected mice had significantly higher frequencies and absolute numbers of *Ehrlichia* Hsp60 _43–63_ and P28-19 _55–75_ specific IFN-γ-producing CD4+ Th1 cells in their spleen (Figure 10).

## Discussion

The genetic diversity of microorganisms presents a challenge in developing broadly effective vaccines [23]. Vaccine design is progressing from empiricism towards the increasingly rational presentation of the targets of protective immunity [1]. Using structural information of proteins, it is possible to engineer optimized antigens that are more stable, homogeneous, and efficiently produced, making immunization more practical and affordable [1]. Thus structural vaccinology is an emerging strategy for the rational design of vaccine candidates [2]. Structural vaccines based on epitopes are more specific, safe, and easier to produce [24], and peptide vaccines based on the epitopes represent a potential strategy for the prevention and treatment of pathogenic diseases, cancers and autoimmune disorders. Their low cost, ease of synthesis and inherent safety are all attractive features [25]. Structure-based design has been employed to develop vaccines against Group B *Streptococcus* infections [2], *Neisseria* [25], and influenza virus [26], [27]. Scarselli et al. [28] combined atomic level structural information with genomics and classical vaccinology to design a single immunogen that elicits protective immunity against more than 300 natural variants of the bacterial pathogen meningococcus group B. This accomplishment provides a glimpse of the power of structure-based vaccine design to create immunogens capable of eliciting protective responses against genetically diverse pathogens.

*Ehrlichia* are host defense-evasive and cell function-manipulative, vector-transmitted pathogens and are responsible for serious diseases of agricultural, veterinary and human importance [8]. *E. chaffeensis, E. ewingii*, and an *E. muris*-like organism are the known species of *Ehrlichia* infecting humans [5], [29], [30]. Interestingly, recent studies demonstrated *E. muris*-like organisms infecting deer in Wisconsin [31]. In dogs, *E. canis*, *E. chaffeensis* and *E. ewingii* are the major pathogens; whereas in livestock of sub-Saharan Africa *E. ruminantium* cause serious disease. As yet there are no commercially available vaccines to protect against ehrlichioses.

Successful trials with live and attenuated vaccines against *E. ruminantium* indicate that vaccine-induced immunity is feasible for *Ehrlichia* spp. containing defined immunoprotective proteins. Some ehrlichial proteins targeted by the host immune response are well defined, such as major OMPs (MAP1, MAP2 and OMP-1) [8]. Studies from our laboratory and others have previously demonstrated the importance of antibodies in protection against *Ehrlichia*. Passive transfer of polyclonal immune sera or mAbs confers protection in SCID mice against *E. muris* and *E. chaffeensis*, respectively [18], [32]. Prior studies in mouse models have demonstrated that vaccination with P28 leads to immunity and clearance of *Ehrlichia* infection.

Using *in silico* techniques, we identified the predicted hydrophilic epitopes of P28-19 and *Ehrlichia* Hsp60 which induce an antibody response. We selected epitope regions which did not have any hydrophobic sequences. Of the three epitopes selected from each of the two antigenic proteins only the peptide corresponding to epitope at the N-terminal sequence of the protein was found to be highly reactive with the sera. One of the reasons the N-terminus sequence of proteins exhibits immunogenicity is that these regions have a higher tendency to be exposed in the protein's native conformation [33]. We also confirmed by BLAST analysis (http://blast.ncbi.nlm.nih.gov/Blast.cgi) that the sequences of P28-19 and *Ehrlichia* Hsp60 selected for peptide generation are highly conserved in the genus *Ehrlichia* (data not shown).

Zhang et al. [34] used recombinant proteins and peptides corresponding to the hypervariable region of P28 to detect *Ehrlichia* specific antibody in infected dogs. In our studies we designed peptides based on hydrophilicity. The P28-19 peptide corresponding to the amino acid sequence 55–75 had the highest reactivity with *Ehrlichia* antibodies, and it also induced antibodies. A previous study showed that mice immunized with the recombinant *E. chaffeensis* P28-19 (rP28) enhanced the spontaneous clearance of the infection in BALB/c mice [35]. A study by Li et al. [18] indicated that a mAb directed against the *E. chaffeensis* P28-19 (OMP-1g) reduced the bacterial burden in SCID mice. Though they reported that the region spanning amino acids 68–90 of P28-19 of *E. chaffeensis* contains a B cell eptiope, subsequent studies by the group [19] utilized the 77–92 amino acid sequence to generate peptides. The peptides were found to be weak in inducing immunogenicity compared to peptides from other regions of P28-19. The high specificity of our peptide may be related to the hydrophilicity of the sequence.

*Ehrlichia* lacks many genes involved in metabolism, and it requires various nutrients and metabolic compounds facilitated through pores or channels in the bacterial outer membrane [36]. Kumagai et al. [36] demonstrated that the *Ehrlichia* outer membrane P28-19 has porin activities and the structure of the protein was consistent with a beta-barrel structure, but the authors did not show the 3D structure of P28. We confirm based on the predicted 3D structure that the P28-19 has a beta-barrel structure and the hydrophilic epitope of our interest is located in the the external loop. Bioinformatics analyses demonstrated that *Ehrlichia* Hsp60 _43–63_ and P28-19 _55–75_ are highly conserved in *Ehrlichia* (>95%). The peptide corresponding to *Ehrlichia* Hsp60 amino acid sequence 43–64 cross reacted with *E. canis* and *E. chaffeensis* antibodies. As the peptides induce an antibody response, we reasoned that the peptide could function as an immunogen, and the induced antibodies could clear the pathogens. Our studies demonstrated that both *Ehrlichia* Hsp60 _43–63_ and P28-19 _55–75_ detected *Ehrlichia* specific antibodies in mice and dogs infected with different *Ehrlichia* species, whereas, the peptides did not react with any non-specific antibodies. A preliminary experiment demonstrated that the peptides could also detect *Ehrlichia* antibodies from human sera infected with *Ehrlichia* (data not shown). The results demonstrated that both the peptides could be used in diagnosis of *Ehrlichia* in animals and humans.

As peptides *per se* are poor in inducing immunogenicity KLH is used as a career protein. It has been demonstrated that use of peptides in ELISA in the absence of KLH career protein was found to be less sensitive [37]. Roberts et al. [37] also demonstrated that KLH was also necessary for a strong and specific antibody response. Vaccination with the *Ehrlichia* Hsp60 _43–63_ and P28-19 _55–75_ peptides did not fully reduce the bacterial load after 7 days of *Ehrlichia* challenge. By day 14 after bacterial challenge there was no detectable *Ehrlichia* load, the antibody levels also increased by day 14 compared to day 7. The low reduction in bacterial load by day 14 may be due to the high levels of antibody and its isotypes on day 14 after bacterial challenge.

Hsps are highly conserved in nature from bacteria to humans [38]. They are molecular chaperones essential for maintaining cellular functions by preventing misfolding and aggregation of nascent polypeptides and by facilitating protein folding [39]. Bacterial Hsp proteins are immunomodulatory and are known to stimulate monocytes and macrophages [40]. Furthermore, Hsps have been reported to be dominant antigens for the host immune response to various pathogens and, thus, have great potential as vaccine candidates [41]. Hsp60 has been an effective subunit vaccine against *Yersinia enterocolitica* [42], and Woo et al. [43] demonstrated that Hsp60 is a highly antigenic protein in *Burkholderia pseudomallei*. Patients infected with *B. pseudomallei* develop a strong antibody response against Hsp60, suggesting that the recombinant protein may be useful for serodiagnosis and vaccine development [43]. Hsp60 of *Francisella tularensis* was evaluated as a vaccine candidate by Noah et al. [44], which elicited secretion of the chemokines CXCL8 and CCL2 through a TLR4-dependent mechanism. A combination of LPS and Hsp60 increased immunity against the pathogen compared to immunization with LPS alone. Analysis of antibody isotypes of mice vaccinated with GroEL of *Salmonella enterica* serovar Typhi suggested predominance of the Th2 type immune response in animals immunized with GroEL and adjuvant, whereas immunization of animals with GroEL-alone shifted the immune response toward the Th1 phenotype. Mice immunized with Hsp60 showed a significant increase in lymphocyte proliferation and had higher IFN-γ and IL-2 levels [40], and demonstrated effective protection by Hsp60 against human typhoid. Hsp60 also provided protection against *B. anthracis* by stimulating both humoral and cell mediated immunity [45].

In addition to their use as indicators of cytokine bias during infection, antibody isotypes have direct functional relevance to disease severity in helminth-malaria co-infection. Antibodies are absolutely required for the ultimate clearance of malaria parasites. In mice, antibodies of the cytophilic isotype IgG2a have been shown to recognize infected erythrocytes and facilitate their destruction by phagocytes. Similarly, in humans IgG1 and IgG3 antibodies are associated with enhanced parasite clearance (reviewed by Fairlie-Clarke et al. [22]).

We observed an increase in spleen size (splenomegaly) after infection (data not shown). The size of the spleen was largest on day 14 and was gradually reduced after day 21, and by day 28 the size corresponded to that of a normal mouse. *E. muris* infection resulted in an increase in antibody levels on day 14 after bacterial infection; interestingly the vaccinated mice also cleared the bacteria completely on day 14. It has been demonstrated that antibodies against *Bordetella parapertussis* have no effect until *B. parapertussis*-specific T cell responses are generated around day 14 [46]. The *Ehrlichia* Hsp60 and P28-19 peptides are likely to induce CD4+ T cell responses as we detected antigen-specific IFN-γ-producing memory CD4+ T cells in mice infected with *E. muris* on day 45 post-infection. Our study suggests *Ehrlichia* Hsp60 _43–63_ and P28-19 _55–75_ peptides controlled *Ehrlichia* by stimulating B and T cells.

Overall, our studies demonstrate the power of structural vaccinology. Peptides designed based on the hydrophilicity of P28-19 and *Ehrlichia* Hsp60 could act as diagnostic antigens and could also function as a vaccine. Rudra et al. [47] demonstrated a self-assembling peptide acting as an immune adjuvant. Future studies will involve the design and development of structural vaccines, which could also include peptide sequences that could act as adjuvants.

## Materials and Methods

### Design of P28-19 and *Ehrlichia* Hsp60 peptides

To determine a protein sequence for potential antigenic epitopes, sequences that are hydrophilic, surface-oriented, and flexible are selected. Most naturally occurring proteins in aqueous solutions have their hydrophilic residues on the protein surface and hydrophobic residues buried in the interior. Three regions of the *E.* muris P28-19 and Hsp60 protein sequence had good hydrophilicity predicted by the Lasergene software (DNAStar, WI, USA). We selected hydrophilic sequences of both the *Ehrlichia* P28-19 and Hsp60 proteins with no hydrophobic residues. The hydrophilic regions of P28-19 correspond to amino acids 55–75, 91–103, and 124–145 (Fig. 1). The hydrophilic regions of *Ehrlichia* Hsp60 correspond to amino acids 43–63, 179–199, and 387–406 (Fig. 2). The sequences showed homology to other *Ehrlichia* species. The peptides (underlined) were synthesized and conjugated to KLH (Biosynthesis, Lewisville, TX) and used as probes to detect antibodies to *E. canis* and *E. chaffeensis* or to raise antibodies.

### 3D structure prediction

The 3D structure of P28-19 in Fig. 1 was modeled using the online I-TASSER (iterative threading assembly refinement) server [48], [49]. I-TASSER builds 3D models from an amino acid sequence using fold recognition and multiple-threading alignments by LOMETS, a meta-threading server in the Zhang lab at the Univ. of Michigan which combines seven state-of-the-art threading programs (FUGUE, HHsearch, MUSTER, PROSPECT, PPA, SP3 and SPARK) then performs iterative structural assembly simulations. The function of the predicted models is then inferred by structurally matching the 3D models with known proteins using protein function databases. The best predicted model from I-TASSER (Fig. 1) gave a C-score of −3.338, a TM-score of 0.34±0.12, and an Exp. RMSD of 14.1±3.8. The C-score is a confidence value for estimating the quality of the model and generally ranges from [−5, 2] with a higher score being better; TM-scores measure structural similarity and are used to measure the accuracy of structural modeling with a TM-score >0.5 indicating a model having the correct topology and a TM-score <0.17 showing random similarity. RMSD is simply the average distance of all amino acid pairs between two structures. Protein segments that are relatively unstructured such a loops and coils can result in high RMSD scores. Based on these results the beta-barrel portion of the model in Fig. 1 is likely to be a reasonable representation of the 3D structure of body of the protein. The coils however, which were modeled *ab initio*, are likely idiosyncratic and there is no way to verify their structure without doing x-ray crystallography or NMR, which are well-beyond the scope of this study. We double checked this model against the best model produced by Phyre2 with similar results. Thus, this model should be approached with caution and care taken not to over-interpret the structure of the loops and coils.

The 3D structure of *Ehrlichia* Hsp60 in Fig. 2 was modeled using the online Phyre2 server [50]. Phyre2 aligns hidden Markov models via HHsearch to improve alignment accuracy and detection rate. In “intensive” mode, which was used here, Phyre2 also incorporates Poing [51], a new *ab initio* folding simulation based on Langevin dynamics, to model regions of the protein that have no detectable homology with known structures. For our Hsp60 sequence, 100% of the residues were modeled at >90% confidence level. The top three PDB models, all GroEL chaperone proteins, had 100% confidence levels and sequence ids of 51–56%. While the model presented in Fig. 2 is likely to be a reasonable estimate of the true 3D structure of this protein, there is no way to validate this so caution should be used in its interpretation.

### Detection of anti-*Ehrlichia* antibodies using the *Ehrlichia* Hsp60 _43–63_ and P28-19 _55–75_ peptides

We used ELISA to detect *Ehrlichia* antibodies in the sera of infected mice and dogs. 250 nanograms of the peptides were coated on an ELISA plate (MaxiSorp, Nunc, Denmark) for 1 hour at room temperature. After washing, the plates were blocked with 5% FCS (in PBS-Tween) for 1 hour. The plates were further incubated with sera of infected mice or dogs for 1 hour at room temperature. Washing was followed by incubation with secondary antibody conjugated to alkaline phosphatase (AP) (Kirkegaard and Perry Laboratories, Gaithersburg, MD) for 1 hour. After the addition of substrates (Blue Phos™ phosphatase substrate, Kirkegaard and Perry Laboratories, Gaithersburg, MD), optical densities were measured using an ELISA plate reader (Molecular Devices, Sunnyvale, CA) at 650 nm after 30 min. incubation at room temperature. All assays were performed in triplicate wells, and the average values were calculated.

### Mice

Six to eight-week old female C57BL/6 mice were used in all experiments. Mice were purchased from the Jackson Laboratory (Bar Harbor, ME) and housed and cared for in the Animal Research Center at the University of Texas Medical Branch in accordance with the Institutional Animal Care and Use Committee guidelines under whose review and approval the experiments were conducted (Protocol No. 95-09-066).

### Immunizations and *Ehrlichia muris* challenge

Mice were immunized i.p., with two doses of 50 micrograms (0.02 µM) of each P28-19 _55–75_ peptide or *Ehrlichia* Hsp60 _43–63_ peptides conjugated to KLH 15 days apart (the first immunization with complete Freund's adjuvant and the second immunization with incomplete Freund's adjuvant) (3 mice per group). Thirty days after the first immunization mice were challenged intraperitoneally (i.p.) with a high dose of *E. muris* (∼1×10^4^ bacterial genomes) and observed daily. Controls included unchallenged naïve mice as well as unvaccinated mice injected with *E. muris* alone. Mice were sacrificed on days 7, 14 and 21 after ehrlichial challenge, and spleen and liver were harvested and sera collected. The ehrlichial load in spleen and liver was determined by quantitative RT-PCR. Sera were assayed for determination of antibody titers.

### Measurement of antibody subclasses

ELISA was performed to measure the concentration of *E. muris*-specific IgG subclass antibodies as described previously [13], [52]. Briefly, the ELISA plates were coated with 50 µl of peptide (*Ehrlichia* Hsp60 _43–63_) or recombinant P28-19 protein at a concentration of 4 µg/ml in PBS. Serum samples were diluted 1∶100, and 100 µl of each sample was added to peptide-coated wells and incubated at 25°C for 1 h. Alkaline phosphatase-conjugated goat anti-mouse IgG1, IgG2c, IgG2b, IgG3, or IgM antibodies (SouthernBiotech, Birmingham, AL) were added at a dilution of 1∶300, and color was developed using Blue Phos™ phosphatase substrate (Kirkegaard and Perry Laboratories, Gaithersburg, MD). Optical densities were measured using an ELISA plate reader (Molecular Devices, Sunnyvale, CA) at 650 nm after 30 min. incubation at room temperature. All assays were performed in triplicate wells, and the average values were calculated. When peptides conjugated to KLH were used as a probe in ELISA, KLH was used as control and the results subtracted from positive values.

### Fluorescence microscopy

P28-19 _55–75_ peptides were injected (i.p. - two times, 15 days apart) into C57BL/6 mice. Antibody was obtained 40 days after the first injection. *E. muris* infected DH82 cells were fixed in 50% methanol-acetone for 5 minutes and later incubated with the anti-*Ehrlichia* P28-19 _55–75_ antibody (or naïve antibody as control) (1∶125) (45 min). After three washes in PBS they were reacted with anti-mouse immunoglobulin G conjugated to Alexa 488. Finally they were mounted in mounting medium containing DAPI (Vectashield, Vector Labs, Burlingame, CA). Experiments were repeated three times. The cells were viewed by epifluorescence microscopy (Olympus BX51, Japan).

### Assessment of ehrlichial load in organs by quantitative real-time PCR

The bacterial burdens in the organs were determined by quantitative real-time PCR. *Ehrlichia*-specific *dsb* gene, which encodes a disulfide bond-forming protein (GenBank accession # AY236484 and AY236485) was selected as the target gene for amplification of *E. muris.* The sequences of the primers and probes and thermal cycle conditions were described previously [15]. PCR analyses were considered negative for ehrlichial DNA if the critical threshold values (Ct) exceeded 40 cycles. Expression of the ehrlichial load was normalized relative to the total DNA (Figure 6). Each sample was run in duplicate.

### Assessment of Hsp60- and P28-19-specific memory CD4+ T cell responses in *E. muris*-infected mice

The frequencies of antigen specific IFN-γ-producing T cells in the spleens were determined by flow cytometric analysis. Splenocytes of individual mice were cultured *in vitro* in a 12-well plate at a concentration of 5×10^6^ cells per well in complete medium (RPMI 1640 medium containing 10% heat-inactivated fetal bovine serum, 10 mM HEPES buffer, 50 µM 2-mercaptoethanol, and antibiotics [penicillin 100 units/ml and streptomycin 100 µg/ml]) in the presence of Hsp60 _43-63_ peptide, P28-19 _55–75_ peptide, recombinant P28-19 or *E. muris* whole cell lysate antigen (5 µg/ml).. Positive and negative control wells contained concanavalin A at a concentration of 5 µg/ml or medium, respectively. The cells were harvested after 18 hours of *in vitro* antigen stimulation (100 microgram per well) followed by 4 hour incubation with Brefeldin A (BD GolgiPlug, BD Biosciences, San Diego, CA) and stained with specific antibodies as described below.

After blocking Fc receptors with anti-Fc II/III receptor mAbs (BD PharMingen, San Diego, CA) in FACS buffer (Dulbecco's PBS without Mg^2+^ or Ca^2+^ containing 1% fetal calf serum and 0.09% sodium azide) at 4°C for 15 minutes, cells were labeled with fluorochrome-conjugated mAbs (BD Biosciences Pharmingen, San Diego, CA) specific for mouse CD3 (APC; clone 17A2), and CD4 (FITC; clone RM4-5), and CD8 (PerCP-Cy 5.5; clone 53–6.7). Later, the cells were fixed, permeabilized and stained for intracellular IFN-γ (PE; clone XMG1.2) using BD Cytofix/Cytoperm Fixation/Permeabilization kit following the manufacturer's instructions. Flow cytometric data were collected using FACSCanto (BD Immunocytometry Systems, San Jose, CA). Live cells were gated based on a vital dye (Near-IR Live/dead fixable dead cell stain; Invitrogen, Carlsbad, CA), and a total of 200,000 events were collected. Data were analyzed using FCS Express software (De Novo Software, Los Angeles, CA). Dot plots were gated on CD3+ T cells and the frequencies and absolute numbers of antigen-specific IFN-γ-producing CD4+ T cells in the spleens were determined after subtracting the background staining of unstimulated cells in wells containing medium only.

### Statistical analysis

When indicated, unpaired two-tailed *t* test was used for comparison of two groups using GraphPad Prism (GraphPad Software Inc., La Jolla, CA). Statistical significance was determined at 95 % (*p*<0.05).
